# Deficiency of Sirtuin 1 Impedes Endometrial Decidualization in Recurrent Implantation Failure Patients

**DOI:** 10.3389/fcell.2021.598364

**Published:** 2021-01-28

**Authors:** Jiaxing Li, Jia Qi, Guangxin Yao, Qinling Zhu, Xinyu Li, Rui Xu, Zhenyi Zhu, Hanting Zhao, Yuan Wang, Ying Ding, Yun Sun

**Affiliations:** ^1^Center for Reproductive Medicine, Renji Hospital, School of Medicine, Shanghai Jiao Tong University, Shanghai, China; ^2^Shanghai Key Laboratory for Assisted Reproduction and Reproductive Genetics, Shanghai, China

**Keywords:** SIRT1, recurrent implantation failure (RIF), decidualization, reactive oxygen species (ROS), endometrial receptivity

## Abstract

Decidualization is driven by differentiation of human endometrial stromal cells (ESCs), and is a prerequisite for successful implantation and establishment of pregnancy. The critical role of impaired decidualization in women suffered recurrent implantation failure (RIF) has been established, while the underlying mechanism is poorly understood. In the present study, we verified the essential role of Sirtuin1 (SIRT1) in regulating differentiation and maintaining reactive oxygen species (ROS) homeostasis of human ESCs during decidualization. The abundance of SIRT1 was decreased in RIF patients both in the endometria during window of implantation phase and in the decidualized ESCs. Downregulation of SIRT1 disrupted the intracellular ROS homeostasis during decidualization of ESC, manifested as the accumulation of intracellular ROS level and the reduction of antioxidant stress molecules. Elimination of ROS with *N*-acetyl-L-cysteine (NAC) could rescued the decidualization inhibition caused by SIRT1 knockdown. Further, we explored the insufficient expression of SIRT1 in ESC affected the deacetylation of forkhead box O1 (FOXO1), and thus inhibited the transcriptional activity of FOXO1. This could account for the dysregulation of intracellular ROS homeostasis during decidualization and decreased expression of decidual markers. Collectively, our findings provided insight into the role of down-regulated SIRT1 in the poor decidual response of ESCs in RIF patients.

## Introduction

Recurrent implantation failure (RIF) refers to the failure of achieving a pregnancy after at least three *in vitro* fertilization (IVF) cycles in which one or two high-grade embryos are transferred (Ruiz-Alonso et al., 2013; Polanski et al., 2014; Bashiri et al., 2018; Zhou et al., 2019). Successful implantation requires a receptive endometrium, a functional embryo and a synchronized communication between maternal and embryonic sides (Neykova et al., 2020). In RIF patients, endometrial dysfunction and poor embryo quality are the most crucial causes of implantation failure (Kliman and Frankfurter, 2019; Patel et al., 2019). Despite there has been great progress in improving embryo quality by advanced assisted reproduction technologies, poor endometrial receptivity is still a bottleneck issue in RIF patients (Davari-Tanha et al., 2016). Endometrium undergoes changes to be receptive to embryos (Lessey and Young, 2019), and during the process decidualization spontaneously occurs. Decidualization refers to the transformation of the endometrial stromal cell (ESC) and differentiation into specialized decidual cells (Gellersen and Brosens, 2014). Decidual cells produce characteristic growth factors and cytokines such as insulin-like growth factor binding protein1 (IGFBP1) and prolactin (PRL), and these biomolecules are used widely as markers of decidualization (Gellersen et al., 2007). Recently, a single-cell analysis of midluteal endometrium identified coding iodothyronine deiodinase 2 (DIO2) and scavenger receptor class A member 5 (SCARA5) as marker genes of decidual response *in vivo* (Lucas et al., 2020). Previous studies demonstrated impaired decidualization in RIF patients, indicating decidualization as an important cause of RIF (Kliman and Frankfurter, 2019; Zhou et al., 2019). However, the molecular mechanisms and potential treatment target underlying impaired decidualization in RIF patients need to be clarified.

Sirtuin 1 (SIRT1), an NAD^+^-dependent deacetylase, senses and modulates the cellular redox status by directly deacetylate key proteins, such as forkhead box O1 (FOXO1) (Zhang et al., 2017). Some researchers observed that SIRT1-forkhead box O (FoxO-) dependent mechanisms could increase the protection against oxidative stress by upregulating key antioxidant enzymes, such as catalase (CAT), superoxide dismutase 2 (SOD2), and peroxiredoxin (Hasegawa et al., 2008; Zhang et al., 2010; Pardo et al., 2011; Hori et al., 2013; Cheng et al., 2014). And SIRT1 maintaining redox homeostasis within the cell is related to nuclear factor-E2 related factor 2 (NRF2), which is an important sensor protein of oxidants to cope with oxidative stress (Huang et al., 2013; Huang K. et al., 2017). Oxidative stress is defined as the increase of reactive oxygen species (ROS) causing pro-oxidant–antioxidant imbalance which interfere cellular function. Among the drastic changes of human ESCs when differentiating, oxidative stress is a potential key factor in decidualization (Ruder et al., 2008; Al-Gubory et al., 2010; Zhu et al., 2014; Owusu-Akyaw et al., 2019). Excessive oxidative stress is thought to represent a common pathological pathway in a spectrum of pregnancy disorders (Sugino et al., 2000; Burton and Jauniaux, 2004; Redman and Sargent, 2005; Wu et al., 2015), thus maintaining cellular homeostasis in the endometrium under oxidative stress is essential for pregnancy (Qin et al., 2016). In fact, decidual cells are extraordinarily adapted to cope with ROS by upregulating free radical scavenger SOD2 (Kajihara et al., 2011; Tamaru et al., 2019) and to prevent oxidative cell death caused by exogenous ROS (Kajihara et al., 2006; Leitao et al., 2010). However, the role of SIRT1 maintaining intracellular ROS balance in the endometrium remains unclear.

In the present study, we demonstrated the possible role of SIRT1 in decidualization defect in ESCs derived from RIF patients. Our primary aim in this study was to clarify the implication of endometrial SIRT1 under-expression on the pathophysiology of RIF. A secondary aim was to elucidate the possible relationship between SIRT1 and ROS in RIF patients, and therefore to emphasize the crucial role of SIRT1 in the differentiation of ESCs.

## Materials and Methods

### Patient Recruitment

Patients were recruited from the Center for Reproductive Medicine, Ren Ji Hospital, School of Medicine, Shanghai Jiao Tong University between July 2018 to July 2019. Written informed consent was obtained, and approval of the ethics protocol was granted from the Ethics Committee of Renji Hospital (2018072608).

The RIF group consisted of patients who had undergone three or more previous failed cycles with at least four morphologically high-grade embryos, but failed to conceive in which there was no other explanation after a through infertility work-up (Ruiz-Alonso et al., 2013; Polanski et al., 2014; Bashiri et al., 2018; Zhou et al., 2019). The control (CTRL) group was women who underwent IVF treatment due to tubal factor infertility, and were able to successfully conceive in first transplantation cycle. All subjects, 24–35 years old, were of Han ethnicity and they all exhibited menstrual regularity with 28–35 days cycles. There is no significant difference in body mass index (BMI) and the basal serum hormonal profiles including follicle-stimulating hormone (FSH), luteinizing hormone (LH), testosterone (T), estradiol (E2) and anti-Mullerian hormone (AMH) between two groups. The demographic features of recruited patients are given in Supplementary Table 1.

### Tissue Collection

The endometrial biopsies were collected with endometrial suction curettes (Runting) from patients undergoing GnRH antagonist stimulation cycle without fresh embryo transfer. The biopsies were obtained on the seventh day after hCG injection which also known as the window of implantation (WOI) phase (Achache and Revel, 2006). The endometrial biopsies from CTRL (*n* = 12) and RIF (*n* = 12) in WOI phase were snap frozen in liquid nitrogen for extraction of messenger RNA (mRNA) and protein. The ESCs were isolated from other patients including CTRL (*n* = 55) and RIF (*n* = 7) in accordance with the protocols described as follows. The CTRL group is women undergoing IVF for tubal factor infertility.

### Immunohistochemical Staining

Protein expression of SIRT1 was assessed in paraffin-embedded endometrial tissue sections. Immunostaining was performed on 4-μm-thick tissue sections. The sections were deparaffinized in xylene (Xylene I 20 min, Xylene II 20 min) and rehydrated through a graded ethanol series (100% alcohol 10 min, 100% alcohol 10 min, 95% alcohol 5 min, 80% alcohol 5 min, 70% alcohol 5 min), and heat induced antigen retrieval used Tris-EDTA buffer (1 mM EDTA, 10 mM Tris, 0.05% Tween 20, pH 9.0) to induced heat antigen retrieval for 20 min. In order to remove activity of endogenous peroxidase, the sections were incubated with 0.3% Triton X-100 for 20 min before blocking with 5% goat serum. The sections were incubated with primary antibody (SIRT1, 1:100, 3 μg/ml, Proteintech, Wuhan, Hubei, China) or without primary antibodies as the negative control overnight at 4°C. After washing with PBS, the sections were incubated with secondary antibody and visualized with 3,3-diaminobenzidine (DAB) and hematoxylin (counterstain). The sections were then counterstained with hematoxylin. Images were obtained with a microscope (Zeiss, Jena, Germany).

### The Isolation of Primary Endometrial Stromal Cell (ESC) and Decidualization *in vitro*

The endometrial tissues were processed for stromal cell culture as described previously (Fernandez-Shaw et al., 1992). Briefly, the samples were suspended in PBS after washing twice in 0.9% normal saline. The tissues were then washed twice in DMEM/F12, chopped and digested with 0.2% collagenase type I (c0130; Sigma, St. Louis. MO, United States) for 50 min at 37°C and finally digested with 0.1% deoxyribonuclease (DN25; Sigma) for 20 min at 37°C. The suspension was filtered through a 40 μm griddle (CLS431750-50EA; Sigma, United States), which permits only stromal cells to pass. Primary ESCs were cultured in Phenol Red-free Dulbecco’s modified Eagle’s medium (DMEM)/F-12 (Gibco, Grand Island, NY, United States) plus 10% charcoal-stripped fetal bovine serum (Biological Industries, US origin) and 1% penicillin-streptomycin-neomycin antibiotic mixture (Thermo Fisher Scientific) at 37°C with a 5% CO2 atmosphere. Cell purity was tested routinely by immunofluorescence staining for cytokeratin and vimentin.

To induce decidualization *in vitro*, 3 × 10^5^ ESCs were incubated with DMEM/F12 containing 2% charcoal-stripped FBS, 1 μmol/L medroxyprogesterone-17-acetate (MPA) (Sigma) and 0.5 mmol/L N6, 20-*O*-dibutyryladenosine cAMP sodium salt (db-cAMP) (Sigma) for 4 days, and the medium was changed every other day.

### Primary ESC Treatment

The treatment of ESCs proceeded simultaneously with artificial decidualization. SIRT1 selective activator SRT1720 (10 μM; Selleck Chemicals) was used to mimic the effect of SIRT1 on decidualization. To further investigate the involvement of ROS in SIRT1-dependent abnormal decidualization, the cells were incubated with or without *N*-acetyl-L-cysteine (NAC) (10mM; Sellect Chemicals, Houston, TX, United States). The treatment of ESCs proceeded simultaneously with artificial decidualization. The medium to induce decidualization for 4 days with or without SRT1720 or NAC was changed every other day. After treatment, total mRNA and protein were extracted for analysis with quantitative real-time PCR (qRT-PCR) or Western blotting. And the cell images were captured with an Olympus microscope.

### Immunofluorescent (IF) Staining

The isolated primary ESCs in the chamber slide (BD Biosciences) were fixed with 4% paraformaldehyde and then permeabilized with 0.4% Triton X-100. After washing, the cells were blocked with normal goat serum (Proteintech, China) for 1 h and then incubated with anti-SIRT1 antibody (1:100, 3 μg/ml, Proteintech) or anti-FOXO1 antibody (1:100, 0.88 μg/ml, Cell Signaling Technology, Danvers, MA, United States) or without primary antibodies as the negative control overnight at 4°C. After washing with phosphate-buffered saline (PBS), the cells were incubated in darkness for 2 h at room temperature with Alexa Fluor 488 (green color)- and 598 (red color)-labeled secondary antibodies (Proteintech). Nuclei were stained with 4′,6′-diamino-2-phenylindole (DAPI) (1 μg/ml).

Immunofluorescence staining of the cytoskeleton was performed according to the protocols of Tubulin-Tracker Red (Beyotime Biotechnology, Shanghai, China). After primary ESC was fixed as mentioned above, 100 μl liquid of Tubulin-tracker Red was added and incubated at room temperature in dark for 40 min. Then PBS containing 0.1%Triton X-100 was used for washing for three times. Nuclei were stained with 4′,6′-diamino-2-phenylindole (DAPI) (1 μg/ml).

All images were obtained with a fluorescence microscope and camera connected to a computer with an image analysis system (Zeiss).

### Small Interfering RNA (siRNA) Transfection

siRNAs (GenePharma, Shanghai, China) against SIRT1(sense: 5′-CCCUGUAAAGCUUUCAGAATT-3′; antisense:5′-UUCUG AAAGCUUUACAGGGTT-3′), FOXO1(sense:5′-GGACAACAA CAGUAAAUUUTT-3′; antisense: 5′-AAAUUUACUGUUGUU GUCCTT-3′) or randomly scrambled siRNA (negative control) were delivered into ESCs using Lipofectamine 3000 Reagent (Invitrogen, Carlsbad, CA, United States) according to the manufacturer’s protocol. For *in vitro* induced decidualization, cAMP and MPA were added after 24 h of transfection for 4 days with the medium being changed every 2 days.

### Extraction of RNA for qRT-PCR

Total RNA from cells and endometrial biopsies were extracted by using a total RNA Kit (Foregene, Chengdu, China) according to manufacturer instructions and was reverse transcribed to cDNA using the PrimeScript^®^ RT kit (Takara, Dalian, China) with appropriate controls. Quantitative real-time PCR was performed and analyzed with ABI Prism System (Applied Biosystems) using SYBR^®^ Premix (Takara) in triplicate. Relative mRNA expression was calculated by the comparative cycle threshold method (ΔΔCt) with β-actin as the housekeeping gene.

The primer sequences used of targeting genes were as follows:

SIRT1(human):5′-TGTGTCATAGGTTAGGTGGTGA-3′ (forward) and 5′-AGCCAATTCTTTTTGTGTTCGTG-3′ (reverse);
FOXO1(human):5′-TGGAGATCGACCCGGACTTC-3′ (forward) and 5′-CCGAGTTGGACTGGCTAAACT-3′ (reverse);
PRL(human):5′-GGAGCAAGCCCAACAGATGAA-3′ (forward) and 5′-GGCTCATTCCAGGATCGCAAT-3′ (reverse);
IGFBP1(human):5′-TTTTACCTGCCAAACTGCAACA-3′ (forward) and 5′-CCCATTCCAAGGGTAGACGC-3′ (reverse);
DIO2(human):5′-ACTCGGTCATTCTGCTCAA-3′ (forward) and 5′-TTCCAGACGCAGCGCAGT-3′ (reverse);
SCARA5(human):5′-CATGCGTGGGTTCAAAGGTG-3′ (forward) and 5′-CCATTCACCAGGCGGATCAT-3′ (reverse);
β-actin(human):5′-CATGTACGTTGCTATCCAGGC-3′ (forward) and 5′-CTCCTTAATGTCACGCACGAT-3′ (reverse).

### Extraction of Protein and Western Blotting

Protein was extracted from cells and endometrial biopsies using ice-cold radio-immunoprecipitation assay lysis buffer (CWBIO, Beijing, China) containing protease inhibitor cocktail and phosphatase inhibitor (Roche, Basel, Switzerland). As for nuclear and cytoplasmic proteins, Nuclear and Cytoplasmic Protein Extraction Kit (Sangon Biotech, Shanghai, China) was used.

After determination of protein concentration by Bradford assay, 30 μg of protein from each sample was electrophoresed in a 10% SDS-PAGE gels and then transferred onto a nitrocellulose blot. After blocking with 5% non-fat milk, the blot was incubated with antibodies against SIRT1 (1:1000; 13161-1-AP; Proteintech), FOXO1 (1:1000; 2880; Cell Signaling Technology, Danvers, MA, United States), Acetyl-FOXO1 (1:200; A17406; ABclonal, Wuhan, Hubei, China), NRF2 (1:1000; ab62352; Abcam, Cambridge, United Kingdom), SOD2 (1:1000; 24127-1-AP; Proteintech). A G-Box iChemi Chemiluminescence image capture system (Syngene) was used to visualize the bands. β-actin (1:10000; 60004-1-Ig; Proteintech) or Lamin A/C (1:2000 dilutions, 4777; Cell Signaling Technology) was used as the loading controls, respectively.

### Co-immunoprecipitation (co-IP)

Co-IP was performed in lysates prepared from pre-treated primary ESCs (120 mg of total protein) using an FOXO1 antibody (Cell Signaling Technology) or normal rabbit IgG (Proteintech) overnight at 4°C with gentle rotation. On the next morning, the protein-antibody complex was incubated with 20 μl of magnetic Protein A + G Beads (Millipore, Billerica, United States) for 1 h at 4°C with gentle rotation. Then, the antibody-protein-bead complex was washed five times with co-IP buffer (Cell Signalling Technology). Then, the protein in the complex was eluted with 20 μl of 1× loading buffer and boiled before running on a 10% SDS-polyacrylamide gel and transferred to a nitrocellulose membrane. After blocking, FOXO1-combined SIRT1 protein was immunoblotted using antibodies against SIRT1.

### ROS Assays

Intracellular ROS level was measured with a ROS Assay Kit (Beyotime Biotechnology, Shanghai, China), according to the manufacturer’s instructions. To detect intracellular ROS, ESCs (2 × 10^4^ cells/mL, 100 μl/well) were seeded in the 96-well plate. After treatment, cells were incubated with 10 μM DCFH-DA probe at 37°C for 20 min. Then cells were washed twice with PBS. The fluorescence was read with a fluorescence microplate reader at excitation/emission of 488/525 nm. The fluorescence images were obtained by a fluorescence microscope.

### Statistical Analysis

Quantitative variables are given as the mean ± SEM. After the examination of normal distribution, *t*-test and one-way or two-way ANOVA analysis of variance followed by a Tukey test for multiple comparisons in Graph Pad Prism software (Graphpad version 5.0) were used where appropriate to assess the differences. Spearman or Pearson correlation analysis was performed to test the correlation between variables after verifying the normal distribution. *P* < 0.05 was considered to be statistically significant.

## Results

### Decreased SIRT1 Abundance in Endometria of RIF Patients

**FIGURE 1:**
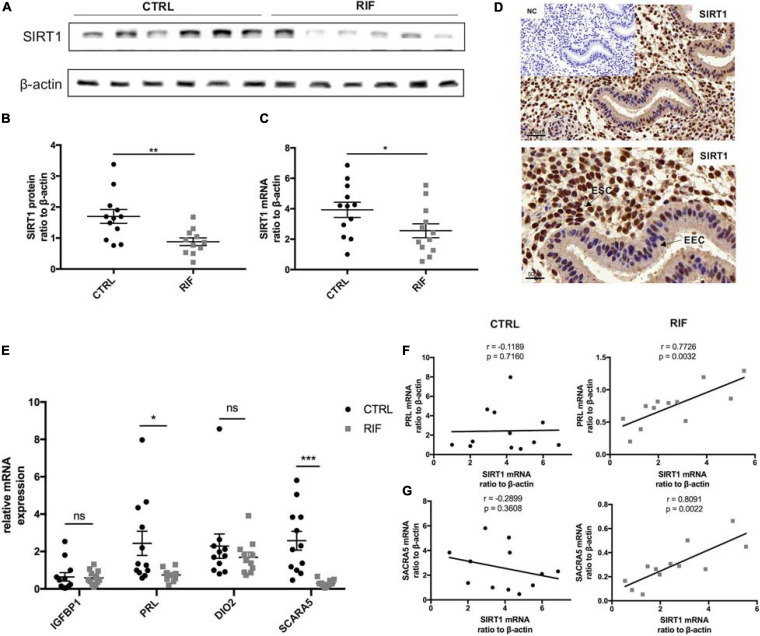
The abundance and location of SIRT1 in the endometrium. **(A)** The representative blot of SIRT1 in endometria from CTRL (*n* = 6) and RIF (*n* = 6). **(B,C)** The abundance of SIRT1 in endometria from CTRL (*n* = 12) and RIF (*n* = 12). The SIRT1 expression levels were normalized to β-actin. **(D)** Immunohistochemical staining of SIRT1 in human uterine endometrium (WOI phrase), the negative control (NC) showed no brown staining. EEC, endometrial epithelial cells; ESC, endometrial stromal cells. **(E)** The mRNA levels of IGFBP1, PRL, DIO2, and SCARA5 in endometria from CTRL (*n* = 12) and RIF (*n* = 12). **(F)** Correlation analysis of SIRT1 and PRL mRNA levels in the endometrium from CTRL group and RIF group. **(G)** Correlation analysis of SIRT1 and SCARA5 mRNA levels in the endometrium from CTRL group and RIF group. ^∗^*P* < 0.05, ^∗∗^*P* < 0.01, and ^∗∗∗^*P* < 0.001 (data are means ± SEM).

Protein and mRNA abundance of SIRT1 in the endometria of RIF patients in WOI phase were significantly decreased in comparison with the control (CTRL) patients (Figures 1A–C). Immunohistochemical staining revealed strong staining of SIRT1 in stromal cells and low-intensity staining in epithelial cells (Figure 1D). Meanwhile, the mRNA level of PRL and SCARA5, two important decidualization biomarkers (Daly et al., 1983; Lucas et al., 2020), was significantly reduced in RIF patients (Figure 1E). A correlation analysis was performed between SIRT1 mRNA levels and PRL or SCARA5 mRNA levels in RIF and CTRL group. The correlation analysis between SIRT1 mRNA levels and PRL mRNA levels in RIF and CTRL group showed the relativity of SIRT1-PRL expression in endometria of CTRL group is weak (*r* = −0.1189, *p* = 0.7160), while there was a significant positive correlation in RIF patients (*r* = 0.7726, *p* = 0.0032) (Figure 1F). Moreover, SIRT1 mRNA level was positively correlated with SCARA5 mRNA level in the RIF group (*r* = 0.8091, *p* = 0.0022), but no significant correlation in the CTRL group (*r* = −0.2899, *p* = 0.3608) (Figure 1G). These data demonstrated the deficiency of SIRT1 in RIF patients and a possible correlation between SIRT1 and decidualization.

### Impaired ESC Decidualization and Decreased Endometrial SIRT1 Expression in RIF Patients

**FIGURE 2:**
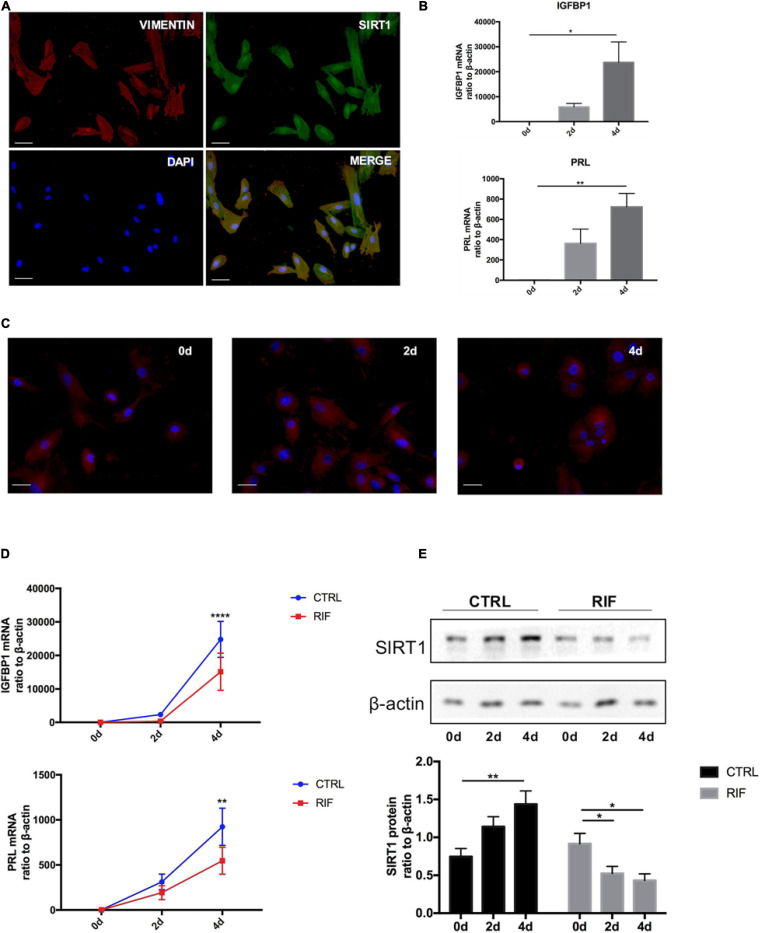
The changes of SIRT1 in decidualization of human ESC. **(A)** Immunofluorescence staining of SIRT1 (green) and vimentin (red) were performed human ESCs. The nuclear were stained with DAPI (4′,6-diamidino-2-phenylindole) (blue). Scale bar is 50 μm. **(B)** The mRNA levels of PRL and IGFBP1 during decidualization (0, 2, and 4 days) *in vitro*. mRNA expression levels were normalized to β-actin. Bar graph was the average data of three independent experiment. **(C)** A cytoskeletal staining of α-tubulin (red) demonstrated differences in cell shape and cytoskeletal architecture. The nuclear were stained with DAPI (4′,6-diamidino-2-phenylindole) (blue). Scale bar is 50 μm. **(D)** The mRNA levels of IGFBP1 and PRL in ESC during decidualization in vitro from CTRL (*n* = 7) and RIF (*n* = 7). **(E)** Western blotting analysis of SIRT1 protein abundance in ESC during decidualization *in vitro* from CTRL (*n* = 7) and RIF (*n* = 7), blots graph was representative and bar graph was the average data. *P < 0.05, **P < 0.01, and ****P < 0.0001 vs. 0 days or CTRL (data are means ± SEM).

The isolated ESCs were identified by immunofluorescence staining with vimentin before further experiments (Figure 2A). Immunofluorescence staining also revealed that SIRT1 was present in nucleus and cytoplasm but mainly localized in nucleus (Figure 2A). Decidualization of ESCs was induced by cAMP combined with MPA *in vitro*. The mRNA levels of two decidual biomarkers (IGFBP1 and PRL) (Gellersen and Brosens, 2003) were notably induced during artificial induction of decidualization (0, 2, 4 days) (Figure 2B). During decidualization, the morphology of ESCs gradually changed from fusiform into polygon-shape (Figure 2C).

To further determine whether there was a decidualization defect in RIF patients, the ESCs were isolated from both RIF and CTRL patients. During artificial decidualization *in vitro*, the expression of decidualization markers (IGFBP1 and PRL) in RIF group was notably inferior to CTRL group (Figure 2D). Unlike in CTRL group, SIRT1 protein level was significantly attenuated in RIF group (Figure 2E) during decidualization *in vitro*. These data suggested that decidualization in RIF patients was disturbed, the expression trend of SIRT1 was inverse during decidualization in RIF patients.

### SIRT1 Is Essential for ESC Decidualization

**FIGURE 3:**
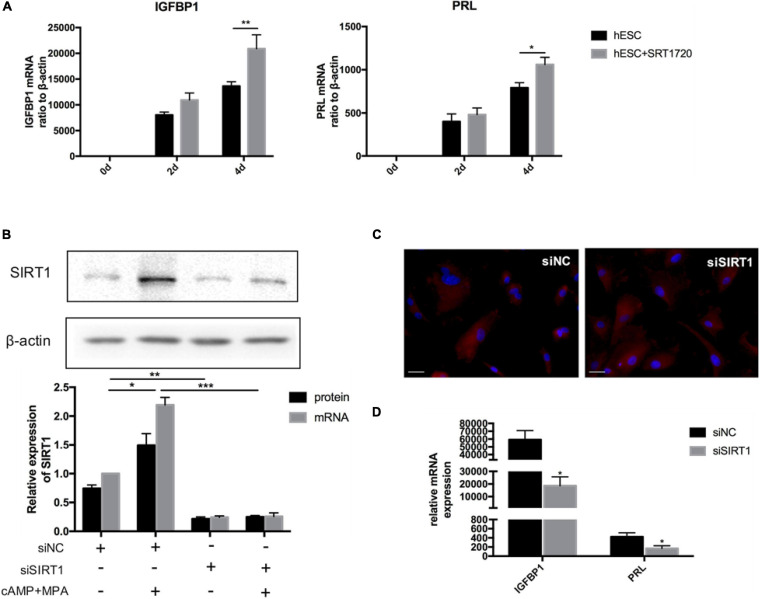
The effect of SIRT1 on ESCs decidualization. **(A)** The mRNA levels of IGFBP1 and PRL following addition of SRT1720 (10 μM) during decidualization (0, 2, and 4 days) *in vitro*. **(B)** Efficiency of siRNA-mediated knockdown in SIRT1 protein abundance and mRNA level. Blots are representative and the bar graphs are the average data of four independent experiment. **(C)** A cytoskeletal staining of α-tubulin (red) demonstrated cellular morphology after 4-day induced decidualization between siNC and siSIRT1 groups. The nuclear were stained with DAPI (4′,6-diamidino-2-phenylindole) (blue). Scale bar is 50 μm. **(D)** The mRNA levels of decidual biomarkers after 4-day induced decidualization between siNC and siSIRT1 groups. mRNA expression levels were normalized to β-actin. Bar graph was the average data of four independent experiments. *P < 0.05, **P < 0.01, and ***P < 0.001 (data are means ± SEM).

When treated with SRT1720, a SIRT1-specific activator, can significantly upregulate the mRNA level of IGFBP1 and PRL on the 4th day of decidualization *in vitro* (Figure 3A). When SIRT1 was silenced (siRNA-mediated knockdown efficiency was more than 80%) (Figure 3B), the mRNA level of decidualization markers (IGFBP1and PRL) were significantly suppressed on the 4th day of artificial decidualization (Figure 3D). We also noted that SIRT1 knockdown caused morphological differences during decidualization: whereas the control cells were enlarged and spherical by induction day 4, the morphology of the siSIRT1-transfected cells remained fibroblast-like (Figure 3C). Therefore, these results supported that deficiency of SIRT1 was detrimental to ESCs decidualization.

### SIRT1-Induced ROS Imbalance Affects ESC Decidualization

**FIGURE 4:**
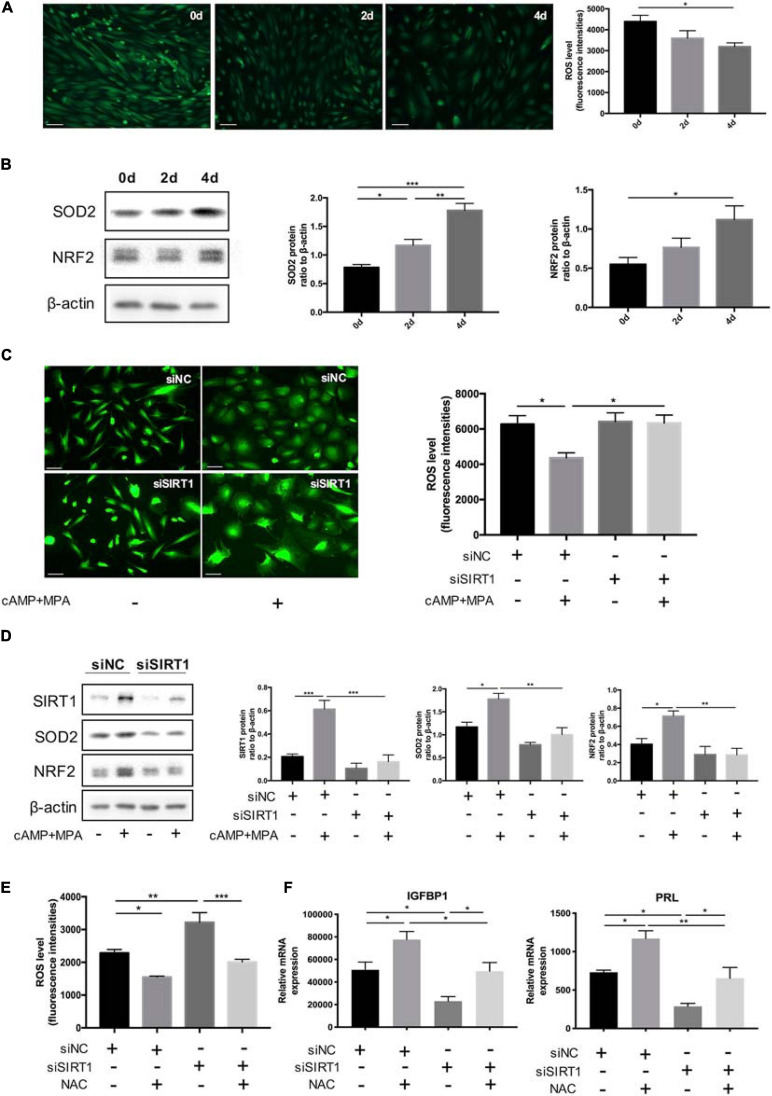
The effect of SIRT1 on ROS homeostasis in ESC decidualization. **(A)** The change of ROS abundance during decidualization *in vitro* (0, 2, and 4 days). ROS levels were measured by the DCFH-DA fluorescence intensities (green). Scale bar is 100 μm. Images was representative and bar graph was the average data of three independent experiments. **(B)** Western blotting analysis of SIRT1, SOD2, and NRF2 protein abundance in decidualization ESC (0, 2, and 4 days). Blots graph was representative and bar graphs were the average data of four independent experiment. **(C)** Quantification of fluorescence intensities of ROS levels between siNC and siSIRT1 groups with and without inducing decidualization treatment. Images were representative and bar graph was the average data of three independent experiments. Scale bar is 50 μm. **(D)** Western blotting analysis of FOXO1, SOD2, and NRF2 protein abundance in siNC, siSIRT1 groups. Blots graph was representative and bar graph was the average data of four independent experiments. **(E)** Quantification of fluorescence intensities of ROS levels after NAC treatment between siNC and siSIRT1 groups. Bar graph was the average data of three independent experiment. **(F)** The mRNA levels of decidual biomarkers with or without NAC treatment in siNC, siSIRT1 groups. mRNA expression levels were normalized to β-actin. Bar graph was the average data of five independent experiment. *P < 0.05, **P < 0.01, and ***P < 0.001 (data are means ± SEM).

The abundance of ROS was decreased in the process of decidualization (0, 2, 4 days) *in vitro* (Figure 4A). Consistently, the protein level of two representative antioxidant, nuclear factor erythroid 2-related factor 2(NRF2) (34) and SOD2(35), were upregulated during decidualization *in vitro* (Figure 4B).

When treated with siSIRT1, the intracellular ROS levels of ESCs were significantly increased after induced decidualization (Figure 4C). Identically, NRF2 and SOD2 were significantly decreased when silencing SIRT1 (Figure 4D). Treatment with antioxidant NAC suppressed the abnormal rise of ROS levels in ESCs caused by knocking down SIRT1 (Figure 4E). NAC could also rescue the defected decidualization. NAC reversed the decline of IGFBP1 and PRL mRNA levels caused by siSIRT1 (Figure 4F). These data suggested that excessive ROS was prejudicial to ESCs decidualization and SIRT1 could modulate ROS abundance in ESCs by affecting ROS clearance.

### SIRT1 Regulated Intracellular ROS Homeostasis Through FOXO1 During ESC Decidualization

**FIGURE 5:**
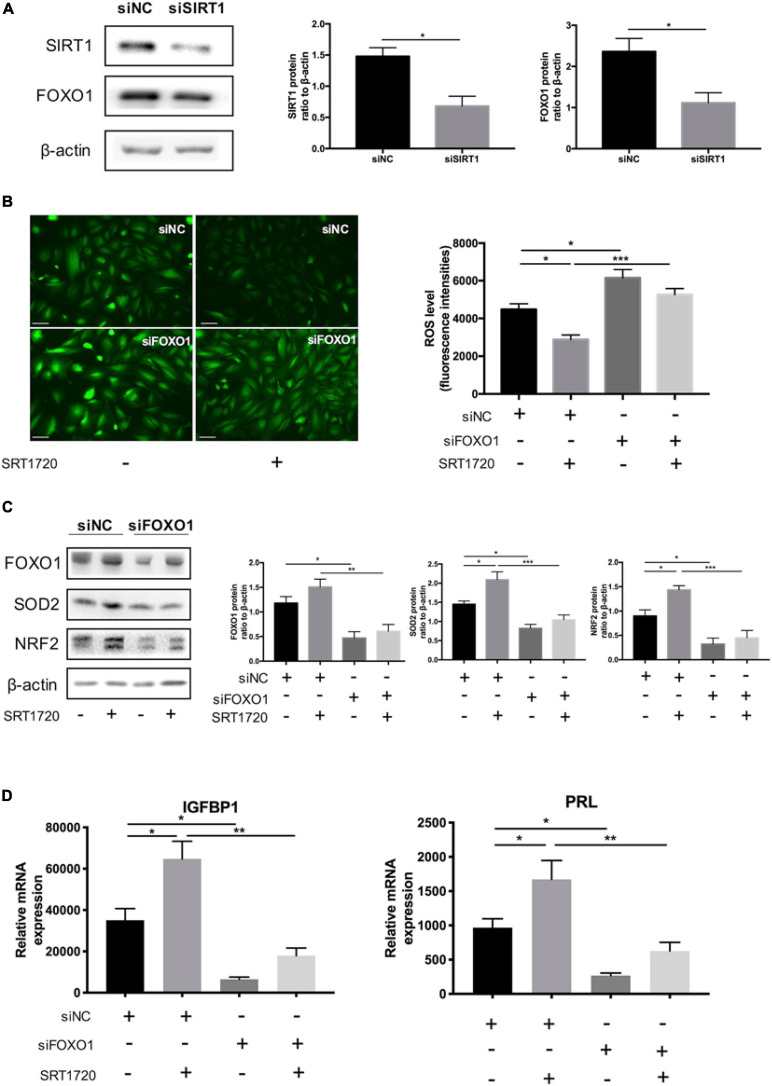
The effect of SIRT1 deficiency on ROS homeostasis through FOXO1 in ESCs. **(A)** Western blotting analysis of FOXO1 protein levels between siNC and siSIRT1 groups. Blots graph was representative and bar graph was the average data of three independent experiments. Scale bar is 50 μm. **(B)** Changes in intracellular ROS abundance between siNC and siFOXO1 groups with or without SRT1720 (10 μM) treatment. Bar graph was the average data of three independent experiments. **(C)** Western blotting analysis of FOXO1, SOD2 and NRF2 protein in siNC, siNC+SRT1720, siFOXO1, siFOXO1 + SRT1720 groups. Blots graph was representative and bar graph was the average data of three independent experiments. **(D)** The mRNA levels of decidual biomarkers in above four groups. mRNA expression levels were normalized to β-actin. Bar graph was the average data of five independent experiments. *P < 0.05, **P < 0.01, and ***P < 0.001 (data are means ± SEM).

FOXO1, an essential transcriptional regulator mediating the expression of IGFBP1 and PRL (Jozaki et al., 2019), has been proved to the acetylation target of SIRT1 (Zhang et al., 2017). Silencing SIRT1 downregulated the protein level of FOXO1 in decidual ESCs (Figure 5A). The data revealed that siRNA-mediated knockdown of FOXO1 could disrupted the role of SIRT1 activator in maintaining endogenous ROS balance: FOXO1 knockdown not only blocked the effect of SRT1720 reducing intracellular ROS levels (Figure 5B) but also attenuated the induction of SOD2 and NRF2 (Figure 5C). At the same time, silencing FOXO1 inhibited the promotion of decidualization of ESCs by SRT1720, based on the expression of decidualization markers (IGFBP1 and PRL) (Figure 5D). These data demonstrated that SIRT1 regulated intracellular ROS homeostasis in ESC decidualization at least in part through FOXO1.

### SIRT1 Affected FOXO1 Translocation by Deacetylation of FOXO1 in Decidual ESCs

**FIGURE 6:**
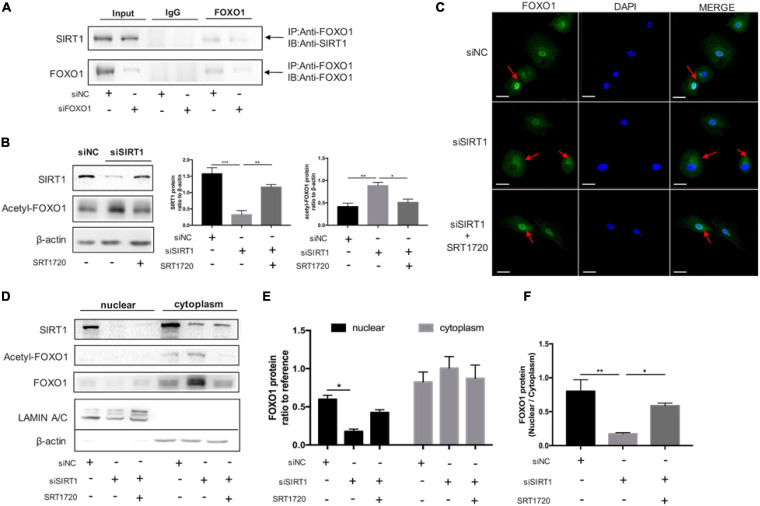
The role of SIRT1 on FOXO1 translocation by deacetylation in decidual ESCs. **(A)** Co-IP assay was used to examine the association between SIRT1 and FOXO1. Input and IgG served as positive and negative controls, respectively. **(B)** Western blotting analysis of SIRT1, Acetyl-FOXO1 abundance after 4-day induced decidualization in siNC, siSIRT1, siSIRT1 + SRT1720 groups. Blots graph was representative and bar graph was the average data of three independent experiments. **(C)** Microscopy images showing immunofluorescence localization of FOXO1 (green) in above three groups; the nuclear were stained with DAPI (blue). Scale bar is 50 μm. **(D)** The representative blot of SIRT1, Acetyl-FOXO1, and FOXO1 proteins in nuclear and cytoplasm of decidual ESCs in above three groups. Blots graph was representative and bar graph **(E)** was the average data of three independent experiments. **(F)** Quantification of the Western blotting assays of FOXO1 distribution and FOXO1 translocation from nuclear to cytoplasm. The nucleoprotein and cytoplasmic protein levels were normalized to Lamin A/C and β-actin, respectively. Bar graph was the average data of three independent experiments. *P < 0.05, **P < 0.01, ***P < 0.001 (data are means ± SEM).

To further investigate the interaction between SIRT1 and FOXO1, we performed Co-IP to confirm SIRT1 could interact with FOXO1in ESCs. The results of the protein interaction analysis confirmed the interaction between SIRT1 and FOXO1 in decidual ESC (Figure 6A). The expression of acetylated FOXO1 was significantly increased when SIRT1 was silenced, and significantly reduced when SRT1720 was added (Figure 6B). Immunofluorescence staining demonstrated that silencing SIRT1 resulted in FOXO1 accumulating in the cytoplasm, and FOXO1 regathered in the nucleus after adding SRT1720 (Figure 6C). Consistently, western blotting revealed that acetylated FOXO1 was weakly expressed in the nucleus (Figure 6D), and silencing SIRT1 could attenuate FOXO1 translocation from cytoplasm to nucleus in ESC while SRT1720 can effectively reverse this process by improving nucleus/cytoplasm ratio of FOXO1 (Figures 6D–F). These data suggested that SIRT1 could improve the nuclear translocation of FOXO1 by deacetylation, and may therefore regulate ESC decidualization by FOXO1 downstream effects.

## Discussion

In this study, we demonstrated for the first time that SIRT1 played an important role in RIF decidualization defect. In RIF patients, the endometrial expression of SIRT1 was decreased and the decidualization was impaired. During decidualization, SIRT1 expression was gradually induced in fertile patients but decreased in RIF patients. Further *in vitro* study in ESCs clarified that insufficient expression of SIRT1 might attenuate decidualization by reducing ROS scavenger enzymes and disrupting the intracellular ROS homeostasis via deacetylation of FOXO1.

RIF remains a critical challenge for clinicians and patients. Suboptimal endometrial receptivity is considered as a key factor in inhibiting embryo implantation in RIF patients (Coughlan et al., 2014). The endometrium is receptive to implantation during WOI phase, which requires epithelial cells develop specialized structures known as pinopodes secreting cell adhesion molecules and stromal cells initiates decidual reactions providing a nutritive and immunoprivileged matrix essential for embryo implantation (Lessey and Young, 2019). Recent years, there has been increased lines of evidence demonstrating that RIF patients suffered from impaired endometrial decidualization (Huang C. et al., 2017; Szwarc et al., 2019; Zhou et al., 2019). Our results also showed that ESCs isolated from RIF patients had a poor response to artificial induced decidualization, manifested by the reduced expression of two decidual markers. Here, we also compared the expression of decidual makers *in vivo*, DIO2 and SCARA5 (Lucas et al., 2020; Tewary et al., 2020), in the endometrial tissues of RIF and control patients during WOI phase. It turned out that PRL and SCARA5 were down-regulated in RIF patients. However, there was no significant difference on IGFBP1and DIO2 which may due to the relative low expression in the initial stage of decidualization instead of senescent decidual cells (Tewary et al., 2020). All the above evidence suggested that the decidualization in RIF patients was defected.

McBurney et al. (2003) found for the first time that SIRT1 null mice are characterized with thin-walled uterus, suggesting that SIRT1 might play an important role in endometrial functions. A study showed RIF patients have higher SIRT1 levels than healthy pregnant women in serum during the 1st to 3rd days of the menstrual cycle, but no statistical significance (Engin-Ustun et al., 2017). However, the abundance of SIRT1 might be different between serum and local endometrial concentrations. In addition, we paid more attention to the level of SIRT1 during the window of implantation phase. Relevant studies have shown that SIRT1 plays an important role in endometrial implantation. A recent research reported that SIRT1 has a positive effect on migration of porcine trophectoderm and uterine luminal epithelium cells, and meanwhile, knockdown of SIRT1 decreased the expression of implantation-related genes (Bae et al., 2020). Preliminary evidence in women would suggest a possible role for SIRT1 in regulating the expression of *E*-cadherin in luminal epithelium, and involved in the initial attachment of embryos (Shirane et al., 2012) but there are also controversial study that have also been concerned (Ling et al., 2020). Our results show that SIRT1 was detected both in endometrial epithelial cells and stromal cells, but the immunohistochemical staining of SIRT1 was stronger in stromal cells. Therefore, the main focus of attention was the role of SIRT1 in decidualization of ESCs.

When refer to the function of SIRT1 in endometrial stromal cells, the results were limited and inconsistent. A previous study reported when using cAMP and P4 for artificial decidualization in ESCs, the expression of SIRT1 was decreased (Ochiai et al., 2019). Ochiai et al. (2019) demonstrated the resveratrol-mediated decidual repression was SIRT1-independent, and the role of SIRT1 on decidualization have not been further explored (Supplementary Figure 2). Our results demonstrated that during artificial decidual induction with cAMP plus MPA, the abundance of SIRT1 was increased (Supplementary Figure 3). The other study also showed the expression of SIRT1 increased after the same method of inducing decidualization, supporting our results (Kajihara et al., 2006). We also found that the expression of decidual markers was significantly suppressed and cell morphology also changed after SIRT1 silencing. Consistently, adding SIRT1-specific agonists could promote decidualization which indicated that SIRT1 silencing and activating could suppress or promote decidualization, respectively. This further verified the essential role of SIRT1 in decidualization. We exclusively explored the contribution of SIRT1 regulating decidualization and endometrial receptivity *in vitro* experiments. Future study requires the utility of conditional knockout mice to more accurately simulate the decidualization process, so as to consolidate the mechanism of SIRT1 in regulating decidualization and endometrial receptivity.

During pregnancy, high levels of ROS were generated in the uterus due to tissue remodeling and profound changes in local perfusion, especially at the fetomaternal interface (Brosens et al., 2009). Previous study suggested that the decidua has heightened ROS scavenging capacity to maintain the integrity of the fetomaternal interface and provide the decidua with a robust system capable of coping with oxidative stress during pregnancy (Sugino et al., 1996; Rytkonen et al., 2019). Our results showed that SOD2 and NRF2, two key scavenger enzymes governed ROS homeostasis, rose while intracellular ROS level decreased during decidualization. Conversely, intracellular ROS level in ESCs increased when knocking down SIRT1, which might be caused by the decreased expression of SOD2 and NRF2. This indicated that SIRT1 is indispensable for decidual ESC to deal with oxidative stress. Moreover, the antioxidant NAC reduced the abnormal intracellular ROS level and eliminated the adverse reactions of ESC decidualization caused by SIRT1 silencing. Our study provides vital preliminary evidence for SIRT1-dependent abnormal intracellular ROS accumulation affecting ESCs decidualization.

Accumulating evidence indicated that SIRT1 deacetylated FOXO1 therefore leading to an upregulation in expression of genes involved in cell-protective processes (Giannakou and Partridge, 2004). Co-immunoprecipitation confirmed the direct interaction between SIRT1 and FOXO1 in the ESC nucleus. Consistent with the previous studies, SIRT1 might regulate mammalian FOXO1 transcription factors through direct binding and/or deacetylation (Yang et al., 2005; Xiong et al., 2011). Here we further clarified FOXO1 was an essential factor in SIRT1-ROS-decidualization signaling. When SIRT1 was selectively activated by SRT1720 in ESCs, the ROS scavenger enzymes were induced while the ROS level was decreased and the decidualization was also enhanced. However, the above active effect of SRT1720 on maintaining ROS homeostasis and enhancing decidualization was completely blocked after knocking down FOXO1. A microarray analysis of FOXO1 knockdown in decidualizing ESCs demonstrated that the induction of SOD2 in response to cAMP and MPA was attenuated (Takano et al., 2007). While to our knowledge the functional relationship between FOXO1 and NRF2 and their capacities to regulate ROS in decidual ESCs have not been studied. Our results showed that knocking down FOXO1 also affects the expression of NRF2, and also blocks the upregulation of NRF2 by SRT1720. Further, we demonstrated that SIRT1 may regulate the expression of SOD2 and NRF2 via FOXO1 deacetylation, to balance intracellular ROS.

In summary, we clarified that the expression of SIRT1 was diminished in the endometrial tissue and ESCs from RIF patients, and the down-regulation of SIRT1 was associated with decidualization disorder. This indicated that a decreased expression of SIRT1 might be a possible cause of decidual defect in RIF. Our *in vitro* study suggested dysregulation of SIRT1 could cause ROS homeostasis imbalance via decreased FOXO1 deacetylation, and therefore caused impaired decidualization. We propose that SIRT1 plays an important role in endometrial decidualization and specific agonist for SIRT1 may provide a potential effective therapeutic approach to the treatment of RIF.

## Data Availability Statement

The raw data supporting the conclusions of this article will be made available by the authors, without undue reservation.

## Ethics Statement

The studies involving human participants were reviewed and approved by Medical Ethics Committee of Renji Hospital, Shanghai Jiao Tong University School of Medicine. The patients/participants provided their written informed consent to participate in this study.

## Author Contributions

JL, JQ, and YS designed the study. JL, HZ, ZZ, XL, RX, YD, and YW collected the patients’ specimens and related information. JL performed the experiments and created the figures. GY and QZ analyzed the data. JL and JQ drafted the manuscript. YS commented on and revised the drafts of the manuscript. All the authors reviewed the results and approved the final version of the manuscript.

## Conflict of Interest

The authors declare that the research was conducted in the absence of any commercial or financial relationships that could be construed as a potential conflict of interest.
