# Supplementary material for: Deficiency of Sirtuin 1 Impedes Endometrial Decidualization in Recurrent Implantation Failure Patients
Source: Front Cell Dev Biol. 2021 Jan 28;9:598364. doi: 10.3389/fcell.2021.598364 (PMC7876093; doi:10.3389/fcell.2021.598364)
Supplement: Supplementary file 1 [file Data_Sheet_1.zip › Supplementary files/Supplementary materials .docx]

**Supplementary Table 1. Demographic features of recruited patients between CTRL and RIF**

|  | CTRL(n=19) | RIF(n=19) | P value |
| --- | --- | --- | --- |
| Age (y) | 29.26 ±2.28 | 29.53±3.08 | 0.77 |
| BMI (kg/m^2^) | 21.77±2.84 | 21.42±2.72 | 0.71 |
| Basal-FSH (mIU/mL) | 6.06±0.94 | 6.08±1.60 | 0.96 |
| Basal-LH (mIU/mL) | 5.13±1.52 | 5.30±2.16 | 0.78 |
| Basal-E2 (pg/mL) | 40.15±15.14 | 41.10±16.87 | 0.86 |
| Basal-T (nmol/L) | 0.97±0.42 | 0.91±0.44 | 0.66 |
| AMH (ng/mL) | 5.14±1.71 | 5.29±2.55 | 0.83 |

All data are Mean ± SEM values.

BMI: body mass index; FSH: follicle-stimulating hormone; LH: luteinizing hormone; E2: estrogen; T: testosterone; AMH: anti mullerian hormone.

**Figure Legends**

**Supplementary Figure 1. The change of PRL concentration after SIRT1 knockdown**

(A) The abundance of SIRT1 between siNC and siSIRT1 groups with and without inducing decidualization treatment. The SIRT1 expression levels were normalized to β-actin. (B) Quantitative of PRL concentration in cultured cell supernatant of endometrial stromal cells between siNC and siSIRT1 groups with and without inducing decidualization treatment. *P < 0.05, **P < 0.01 and ***P < 0.001 (data are means ± SEM from three experiments).

**Supplementary Figure 2. The effect of resveratrol on ESCs decidualization.**

(A) Detection of endometrial stromal cell viability after adding resveratrol with different concentration. (B) The mRNA levels of IGFBP1 and PRL in ESC during decidualization after adding resveratrol with different concentration. **P < 0.01 and ****P<0.0001 (data are means ± SEM from three experiments).

**Supplementary Figure 3. The change of SIRT1 during decidualization**

(A-B) Quantification of the Western blotting assays of SIRT1 in endometria during decidualization (0d, 2d, 4d, 6d, 8d, 10d) in vitro. Blots graph was representative and bar graphs were the average data. **P < 0.01 (data are means ± SEM from three experiments).

**Supplementary** **Materials and Methods**

**Cell viability assay**

The ESCs were seeded in 96-well plates at a concentration of 1× 103 Cells/well and incubated at 37 °C. CCK-8 assay was employed to assess the viability of the cells. After being subjected to the above-mentioned treatments, the cells were washed with phosphate-buffered saline (PBS), and 10ul CCK-8 solution at 10% dilution was added to each well, and the plate was then incubated for approximately 24 h in an incubator. The absorbance at 450 nm was assayed using a microplate reader (Molecular Devices, Sunnyvale, CA, USA). The mean of the optical density (OD) of 3 wells in the indicated groups were used to calculate the percentage of cell viability according to the following formula: cell viability (%) = (ODtreatment group/ODcontrol group) × 100. The experiment was repeated 5 times.
